# sPinal cOrd neUromodulatioN to treat Cerebral palsy in pEdiatrics: POUNCE Multisite Randomized Clinical Trial

**DOI:** 10.3389/fnins.2023.1221809

**Published:** 2023-07-26

**Authors:** Kristin Girshin, Rahul Sachdeva, Richard Cohn, Parag Gad, Andrei V. Krassioukov, V. Reggie Edgerton

**Affiliations:** ^1^GirshinPT, Rancho Cucamonga, CA, United States; ^2^SpineX Inc., Los Angeles, CA, United States; ^3^International Collaboration on Repair Discoveries, Department of Medicine, University of British Columbia, Vancouver, BC, Canada; ^4^Independent Consultant, Chapel Hill, NC, United States; ^5^Spinal Cord Program, GF Strong Rehabilitation Centre, University of British Columbia, Vancouver, BC, Canada; ^6^Rancho Research Institute, Downey, CA, United States; ^7^USC Neurorestoration Center, University of Southern California, Los Angeles, CA, United States; ^8^Institut Guttmann, Hospital de Neurorehabilitació, Institut Universitari adscrit a la Universitat Autònoma de Barcelona, Badalona, Spain

**Keywords:** spinal cord neuromodulation, noninvasive stimulation, cerebral palsy, sensorimotor function, spasticity

## Abstract

**Introduction:**

Cerebral palsy (CP) affects up to 4 children in 1,000 live births, making it the most common motor disorder in children. It impairs the child’s ability to move voluntarily and maintain balance and posture, and results in a wide range of other functional disorders during early development impairments in various sensory modalities, e.g., vision, hearing ability and proprioception. Current standard of care therapy focuses on symptom management and does not mitigate the progression of many of these underlying neurological impairments. The goal of this trial is to conduct a prospective multicenter, double-blinded, sham-controlled, crossover, randomized control trial to demonstrate the safety and efficacy of noninvasive spinal cord neuromodulation (SCiP™, SpineX Inc.) in conjunction with activity-based neurorehabilitation therapy (ABNT) to improve voluntary sensorimotor function in children with cerebral palsy.

**Methods and analysis:**

Sixty participants (aged 2–13 years) diagnosed with CP classified as Gross Motor Function Classification Scale Levels I-V will be recruited and divided equally into two groups (G1 and G2). Both groups will receive identical ABNT 2 days/wk. G1 will initially receive sham stimulation, whereas G2 will receive therapeutic SCiP™ therapy for 8 weeks. After 8 weeks, G1 will cross over and receive therapeutic SCiP™ therapy for 8 weeks, whereas G2 will continue to receive SCiP™ therapy for another 8 weeks, for a total of 16 weeks. Primary and secondary outcome measures will include Gross Motor Function Measure-88 and Modified Ashworth Scale, respectively. Frequency and severity of adverse events will be established by safety analyses.

**Ethics and dissemination:**

The trial is registered on clinicaltrials.gov (NCT05720208). The results from this trial will be reported on clinicaltrials.gov, published in peer-reviewed journals and presented at scientific and clinical conferences.

## Background

Cerebral palsy (CP) is the most common childhood motor disorder, with an estimated 1 in 345 children identified with CP within the United States. It has a global incidence of 17 million and approximately 800,000 individuals are currently living with CP in the U.S. (Oskoui et al., 2013). CP results in weakness, delayed motor development, significant motor impairment ranging from weakness in one hand, to an almost complete lack of voluntary movement in all four limbs. The most common presentation of CP is spastic CP, which is diagnosed in early childhood (Novak et al., 2017; te Velde et al., 2019). However, CP is a lifelong debilitating condition resulting from permanent damage to the developing brain (Flett, 2003). Even though the initial abnormal brain development resulting in CP is not progressive, the cascade of motor impairments that evolve during childhood persists and can worsen into adulthood, reducing the quality of life of the affected individuals and their family members/caregivers (Frisch and Msall, 2013). While the specific causes of CP are often unknown, changes in blood flow, maternal infections, intrauterine stroke, injury, or genetics are common causes. Compared to typically developing children, the medical care costs are 10 times higher for the children with CP, with the estimated lifetime care costs for an individual with CP exceeding $1 million (Kruse et al., 2009).

Currently, there are no treatments for CP that mitigate the underlying neurological dysfunction to enable voluntary movement. The treatment regimen is often individualized, depending on specific needs, severity of symptoms, and affected body parts. Regardless, a primary standard of care tenet for most individuals is activity-based neurorehabilitation therapy (ABNT), potentially with subsequent medication to manage pain and spasticity and/or surgery to relieve symptoms of spasticity. Surgical procedures such as selective dorsal root rhizotomy (SDRs) are often successful in decreasing spasticity, but has limited impact on recovery of voluntary sensorimotor function (Tedroff et al., 2015). Although early intervention with aggressive treatments may promote functional recovery via symptom management, they cannot reverse the neurological damage. Therefore, there is an unmet clinical need in the current clinical care, which is primarily focused on management of symptoms and maintenance of the persistently impaired functional states.

## Proposed solution

To address this need, SpineX Inc. has developed a noninvasive spinal neuromodulation device SCiP™ (Spinal Cord Innovation in Pediatrics), a Class II, Nonsignificant Risk Device, recognized with a breakthrough device designation by US FDA for treating the underlying condition associated with CP. SCiP™ directly modulates the spinal cord neural circuits and indirectly the supraspinal neural circuits by transcutaneously delivering novel electrical pulses below motor thresholds to neuromodulate spinal and supraspinal networks into an activated state of plasticity. Although the exact mechanisms by which transcutaneous electrical spinal neuromodulation transforms the spinal neural networks into more functional states is not fully understood, considerable insights have been gained from studies of spinal cord injury and other forms of paralysis (Edgerton et al., 2021). Our initial findings demonstrated that acute (single session) neuromodulation with SCiP™ in children with CP significantly improved the postural and locomotor abilities in 11 out of the 12 participants (Gad et al., 2021).

In the chronic approach, we combined neuromodulatory (SCiP™) and rehabilitative (ABNT) strategies (Hastings et al., 2022). In our pilot trial, 16/16 children (age 2.5–16) who received SCiP™ therapy in conjunction with ABNT for 8 weeks, demonstrated significant motor recovery. Their Gross Motor Function Measure-88 (GMFM88) scores improved by 13.3 ± 1.3 points (*p* < 0.05), more than twice the minimal clinically important differences (MCID) of 5 points (Storm et al., 2020), and greater than other available treatment options. Functional improvements persisted even in the absence of active stimulation, supporting our working hypothesis that spinal neuromodulation facilitates targeted spinal-supraspinal neural plasticity needed for long-term recovery.

Our hypothesis is that first we neuromodulate the neural networks to an elevated state of plasticity. The second stage of the transformation is to provide activity dependent mechanisms to guide the transformation of the neuromodulated networks to more functionally competent states, depending principally on specific activity dependent guidance derived from specific patterns of proprioception. All data to date from studies from individuals with severe spinal cord injuries (Shackleton et al., 2022) and from our initial studies of CP (Hastings et al., 2022) are consistent with this hypothesis. The potential for neural plasticity, theoretically and with a considerable number of scientific studies (Tang et al., 2019), suggests that there is greater plasticity at younger age (Johnston, 2004). Thus it is imperative to consider the potential advantage of timely and adequate intervention for maximal efficacy in this population. Another factor that shapes the design of the proposed trial protocol is the extreme heterogeneity in severity of neurological impairment in CP, which produces differential functional capabilities within these children. For instance, children with Gross Motor Function Classification System (GMFCS) levels I, II and III are capable of performing tasks such as dynamic standing, overground walking, treadmill stepping and backward walking, whereas children with GMFCS levels IV and V require significant external assistance and training in sitting, trunk and postural activities with minimal treadmill stepping and no backward or overground stepping. Therefore, the ABNT protocol needs to be tailored to accommodate the functional capabilities of these children.

## Trial objectives

The primary objective is to assess the safety and efficacy of 8 weeks of SCiP™ therapy during ABNT compared to sham (inactive) neuromodulation during ABNT in improving voluntary sensorimotor function measured on the GMFM88 scale in children with CP. The secondary objective is to assess the safety and efficacy of short-term (8 weeks) vs. long-term (16 weeks) SCiP™ in conjunction with ABNT in improving voluntary sensorimotor function (GMFM88 score). We hypothesize that children who undergo SCiP™ neuromodulation therapy in conjunction with ABNT will demonstrate a statistically greater improvement in voluntary sensorimotor function as assessed by GMFM88 scores, compared to subjects undergoing sham modality in conjunction with ABNT. The proposed clinical trial was discussed with the US FDA via a virtual sprint discussion owing to the breakthrough device status; and SpineX and the agency have reached an alignment on the clinical trial design, primary and secondary outcomes, sample size and safety outcomes.

## Methods and analysis

### Study design and setting

The trial overview is in accordance with SPIRIT guidelines (Chan et al., 2013), and is illustrated in Table 1. Study participants, their parents/caregivers and physical therapists were involved in study design, choice of ABNT and design of SCiP™ device. The study is designed in three phases:

- Phase I: a primary prospective, multicenter, double blind, two-arm, randomized (1:1) sham controlled clinical study through 8 weeks.
- Phase II: Upon completion of the randomization phase, subjects have the option to enter a secondary non-blinded observation treatment extension phase through 16 weeks.
- Follow-up phase: All patients will be reassessed 8 weeks after the last stimulation session as a follow-up to assess the level of functionality retained.

**Table 1:** The schedule of enrolment, interventions, and assessments based on SPIRIT guidelines.

Activity	Phase I				Phase II		Follow-up
	Screening	Baseline Visit 0	Visits 1 to 16	Primary eval. visit 17	Optional extension visits 18–33	Extension eval. visit 34	Follow-up eval. visit 35
Informed consent	X						
Eligibility assessment	X						
Physical examination	X						
Vital sign assessment	X	X	X	X	X	X	X
Medical history, demographics, medication history	X						
Concomitant medications	X	X	X	X	X	X	X
GMFM88		X		X		X	X
MAS		X		X		X	X
10MWT (GMFCS levels I-III only)		X	X	X	X	X	X
GMFM88 dimension B (GMFCS levels IV-V only)			X		X		
PBS		X		X		X	X
PEDI-CAT		X		X		X	X
PAICP	X	X	X	X	X	X	X
Randomization		X					
Electrode placement; identification of stimulation parameters		X					
Stimulation or sham with ABNT			X		X		
Adverse events		X	X	X	X	X	X
Device deficiencies		X	X		X		
User experience questionnaire				X		X	X
Bang blinding assessment				X			

Up to 10 investigational sites will participate in the study. Investigational sites will be identified and selected based on qualifying criteria, including the volume of targeted subjects who would meet eligibility for study inclusion. No site will contribute more than 35% of the total number of randomized subjects (included in the analysis set). ABNT, Activity Based Neurorehabilitation Therapy; GMFM88, Gross Motor Function Measure; MAS, Modified Ashworth Scale; 10MWT, 10 Meter Walk Test; PBS, Pediatric Balance Scale; PEDI-CAT, Pediatric Evaluation of Disability Inventory Computer Adaptive Test; PAICP, Pain Assessment in Cerebral Palsy.

**Figure 1:**
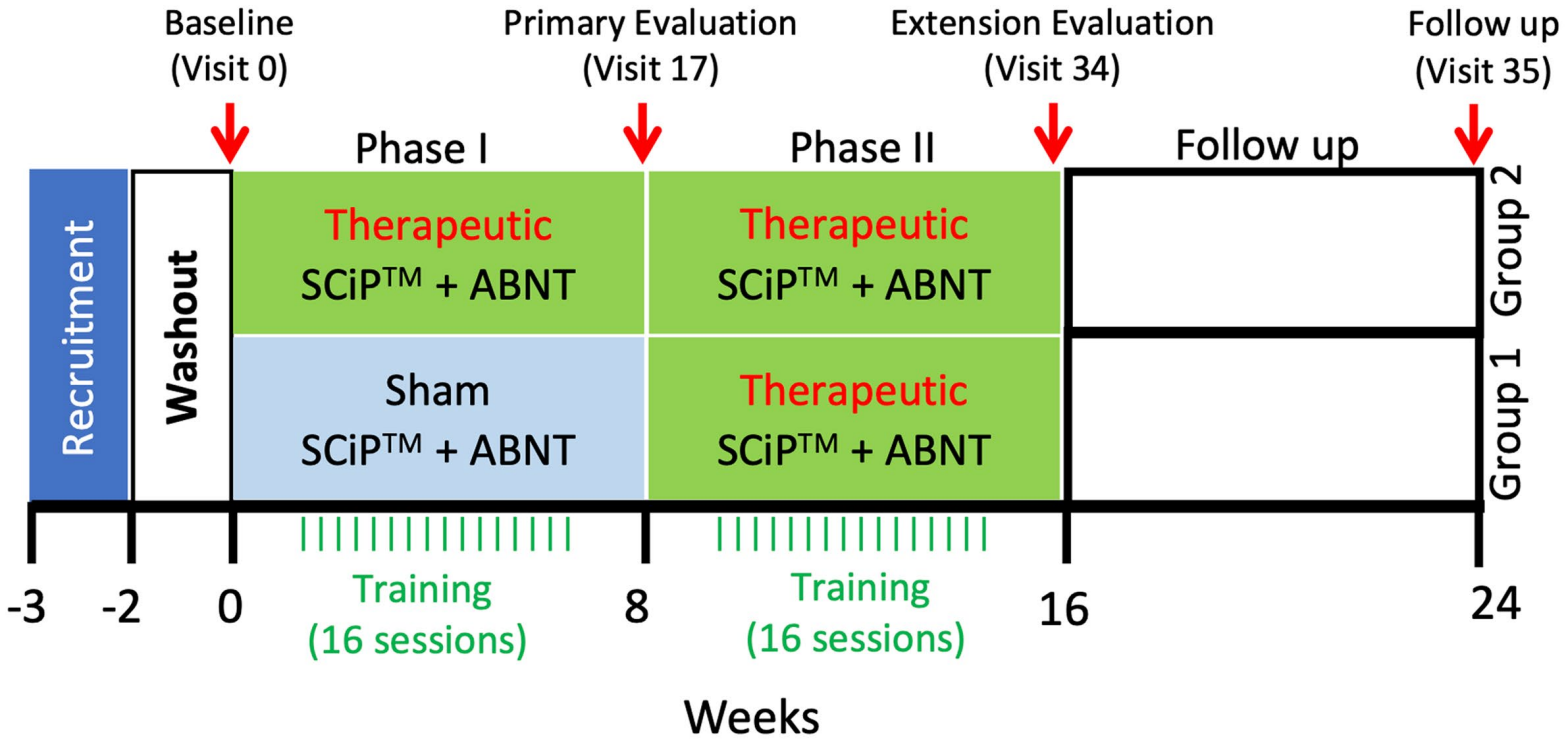
Clinical trial timeline. Following a 2 week washout period, the treatment and sham groups receive therapeutic stimulation or sham stimulation, respectively, for 8 weeks. After the primary evaluation on visit 17, sham group will cross over to begin 8 weeks of therapeutic stimulation, similar to the treatment group that will continue with another 8 weeks of therapy for a total duration of 16 weeks. Following the extension evaluation at visit 34, the participants will receive no intervention for 8 weeks. After 8 weeks of no intervention, participants will return to the clinic for a follow-up evaluation at visit 35 (SCiP ™, Spinal Cord Innovation in Pediatrics; ABNT, Activity Based Neurorehabilitation Therapy).

Children will be observed for up to approximately 30 weeks. This includes an initial washout period of 2 weeks, Screening visit, a Baseline (Visit 0), Phase I (Visits 1–16 of approximately 60-min therapy sessions twice a week for 8 weeks), a Phase I post 8-week evaluation (Visit 17), Phase II (Visits 18–22), an 8-week treatment extension in which the sham group will receive treatment and treatment group will receive an additional 8 weeks of treatment, a Phase II post 8 or 16-week evaluation (Visit 34) occurring within 1–3 days of the last session, and a follow up evaluation 8 weeks post last intervention (visit 35, at week 24). During the washout period, all children will be asked to stop all antispastic medications and other forms of therapies including hippo therapy, intensive therapies etc. A clinical trial outline has been described in Figure 1.

### Study population and recruitment

Up to 80 participants (ages ≥2 and ≤ 13 years), diagnosed with CP, spastic diplegia, spastic hemiplegia or spastic quadriplegia, and GMFCS Level I-V will be consented to achieve a minimum of 60 evaluable datasets (30 treatment, 30 control) at the primary Phase I endpoint of 8 weeks (see statistical analysis for sample size calculation). The inclusion/exclusion criteria are described in Supplementary Table S1.

### Randomization and blinding

Participants will be randomized into a treatment (SCiP™) arm and control (sham) arm in a 1:1 ratio. A block randomization technique will be employed, and randomization will be stratified by age (2–7 and 8–13) and GMFCS level (levels stratified as I + II + III & IV + V; ambulatory and non-ambulatory groups). All participants and the clinicians administering the assessment will be blinded to the initial group assignment. The sham device will be physically identical and intensity on the LCD display will be shielded from the participants. At Phase I primary evaluations, participants will be asked what treatment they believe they received using the Bang blinding index (Bang et al., 2004). Participants will not be blinded during the Phase II, open label extension during which all subjects will receive SCiP™ treatment.

### Intervention

**Figure 2:**
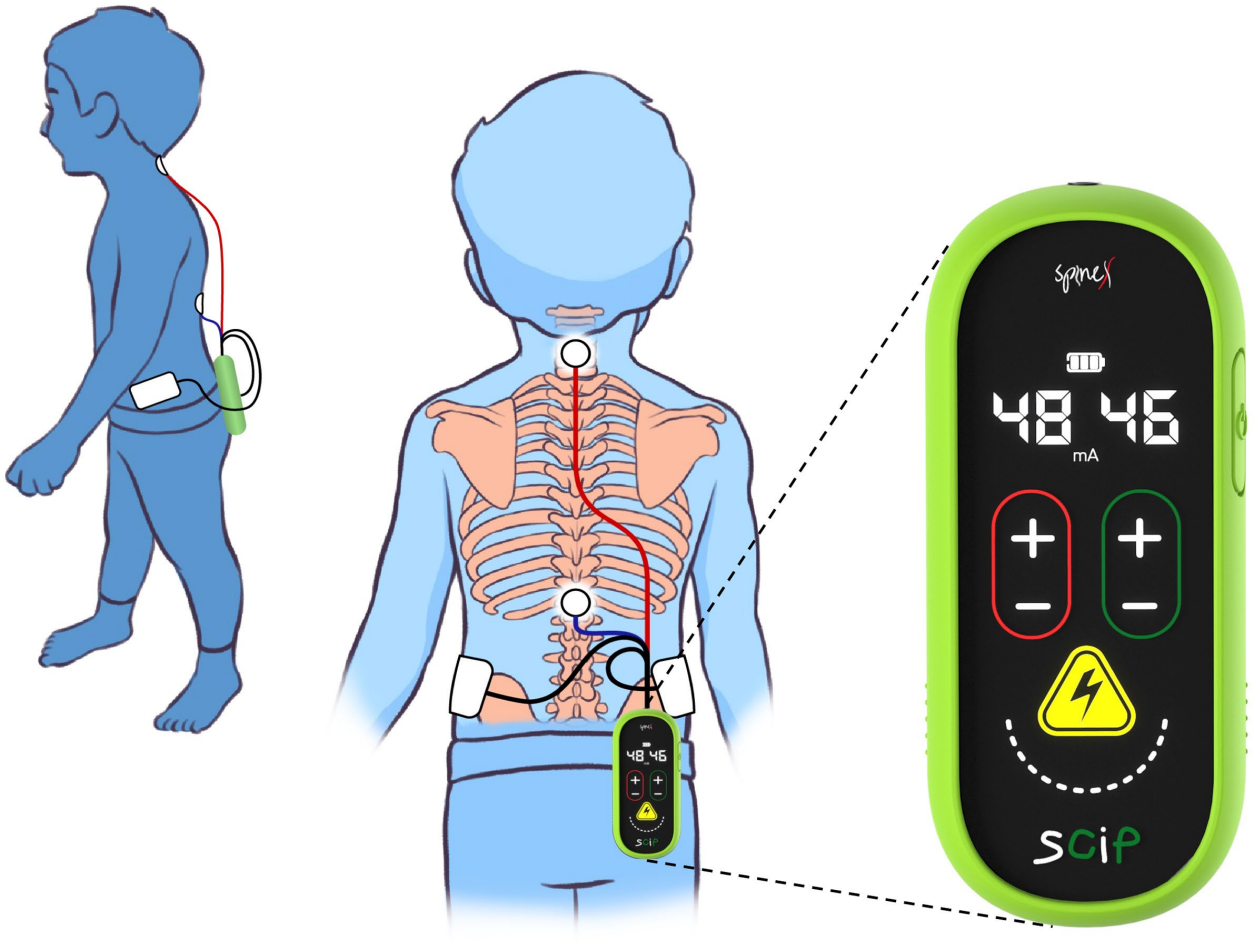
Depiction of wearability and form-factor of Spinal Cord Innovation in Pediatrics (SCiP™) unit and electrodes placement on a child.

The SCiP™ device is a non-invasive electrical neuromodulation device designed to provide transcutaneous spinal cord neuromodulation through the skin (Figure 2). SCiP™ consists of (1) SCiP™ Device, (2) one electrode cable assembly, and (3) four electrodes (one circular electrode placed on cervical, 1.25″ diameter, Axelgaard) and thoracic spine (1.25″ diameter, Axelgaard) and two rectangular electrodes on the hips (2 × 4″, Axelgaard). SCiP™ device uses advanced waveforms that emit a lower overall power density compared to contemporary devices. This allows the pain-free administration of electrical stimulation directly to the spinal cord neural networks (cervical levels C5-6 and thoracic levels T11-12), in order to modulate the circuitry that controls sensorimotor functions of the upper limbs, trunk and lower limbs.

Participants randomized to the therapeutic group will receive transcutaneous neuromodulation via SCiP™ device. The motor threshold will be determined by the therapist via a visible motor contraction of paraspinal, abdominal, upper or lower extremity muscles induced by the stimulation. During training, a sub-motor threshold intensity will be used to ensure no motor evoked responses are generated (muscle contractions) and that the stimulation does not result in pain or discomfort. The stimulation will begin at 0 mA and will be increased by 1 mA every 10 s within sub-motor threshold intensity. Intensities over the C5-6 spine will range between 6 and 20 mA and over the T11-12 between 5 and 16 mA based on the activity being performed and subject-specific responsiveness. Initial stimulation amplitude will be set when the participant voluntarily attempts to extend the cervical or thoracic regions while in a seated position. Based on our preliminary observations, the amplitude will be lowered by 1–2 mA during activities involving sitting, rolling etc., and increased by 2–4 mA during standing and stepping. The intensities will be modulated ±2 mA based on the functional performance of the participant and the sensory perception skills of the therapist during the task.

Participants randomized into the sham group will not receive active electrical stimulation. However, to ensure sufficient blinding, during each session, with the child in a seated position, stimulation will be delivered at C5-6 and T11-12 at an intensity of 1 mA for a period of 1 min. After 1 min, the stimulation will be automatically decreased to 0 mA, however, the SCiP™ device will continue to make a beeping sound mimicking a therapeutic stimulation session. To ensure consistency across multiple sites, all site personnel will be thoroughly trained and provided with a written guidebook.

The participants in both groups will receive ABNT, which is designed based on our preliminary investigations (Hastings et al., 2022), i.e., input from expert pediatric physical therapists, the severity of impairment, acceptability of the subject and caregiver and collaboration with participating study centers. This was done to ensure the uniform inclusion of current standard of care practices and accommodation functional capabilities of individual child. Table 2 describes various ABNT activities based on the GMFCS levels. During ABNT, the goal will be to maintain appropriate posture, midline orientation and head position with center of mass over base of support for optimized weight bearing to organize proprioceptive information. ABNT sessions will be video recorded and analyzed by the contract research organization to ensure consistent delivery of stimulation protocols.

**Table 2:** Description of the ABNT activities based on the GMFCS levels.

#	Approx. time (mins)	Step	GMFCS level	Activity description
**1**	10	Treadmill stepping	All	At speeds from 0.1 to 3.0 mph. Start at slowest speed (0.1 mph), for 3 min, then try to get as high as the child can move their legs. Note: Therapist may need help to advance and to encourage child’s self-initiation. Body weight support (Harness) will be used as needed
**2**	5	Assisted backwards treadmill walking	All	At speeds from 0.1 to 0.3 mph. Therapist may need help to advance and to encourage child’s self-initiation. BWS (Harness) will be used as needed
**3**	5	Side-stepping over level ground	I-III	Have child positioned near wall or a waist high table and perform and activity incorporating side-stepping. Children should take at least 3–4 steps in each direction keeping hips and feet pointed toward wall or table
**3**	5	Sitting: trunk posture and head control	IV-V	Have the child be seated on a bench with the heel on the ground. Their trunk should be maintained in alignment with head, shoulders, and hips in one line.
**4**	5	Functional play running	I-III	Have child run by playing a game that has them run fast and then immediately stop when a verbal cue is given (e.g., games such as Red Light, Green Light, or Mother May I?)
**4**	5	Rolling	IV-V	Have the child in supine position on a mat. Introduce a toy and encourage the child to reach over to that side with their hands and legs
**5**	10	Sit-to-stand transitions and reaching	All	Activity involves an upper extremity activity (usually reaching for a toy) which requires the child to stand. Both hands should be used equally with one-hand and two-hand activities
**6**	10	Jumping and climbing with descending	I-III	Have child ascend and descend 6-inch steps using only legs with therapist assisting as needed.
**6**	10	Half kneel to stand	IV-V	(1) Position the child on all fours (quad kneeling) (2) Child should transition from half knee to standing. They may use their hands on a surface if needed.
**7**	5	Treadmill walking	All	Have child walk on treadmill with good form (i.e., head, hips and heel in one vertical line and heels touching the treadmill belt during initial contact) at slowest speed (0.1–0.3 mps) either independently or the least amount assistance

All participants will be required to discontinue, for 14 days prior to consent and for the duration of their participation in the study, other ongoing physical and occupational therapies, including but not limited to robotic therapy, gait training, aqua therapy, hippo therapy, intensive therapies, whole body vibration, and electrical stimulation therapies.

### Primary outcomes

All assessments will be performed by a trained pediatric physiotherapist who will evaluate all participants at the site. The therapist will be thoroughly trained during the site initiation visit and provided with an illustrated guidebook to ensure consistency across sites. The assessing therapist will be blinded to the group assignments.

Gross Motor Function Measure-88 (GMFM88): GMFM88 is a standardized observational instrument designed and validated to measure change in gross motor function over time in children with CP using a 4-point Likert scale for 88 items across five dimensions: (a) lying and rolling, (b) sitting, (c) crawling and kneeling, (d) standing, and (e) walking, running, and jumping. The participant will complete a number of gross motor activities, depending on the age and ability (Russell et al., 2002; Ko and Kim, 2013). The GMFM88 item scores will be summed to calculate raw and percent scores for each of the five dimensions and a total GMFM88 score. Participants will be designated as responders if they demonstrate at least 5-point increase in GMFM88 score from baseline, as assessed at the end of the 8-weeks (visit 17). Participants will be designated as non-responders if this criterion is not met. The criteria for success are defined as at least 50% of the children in the therapeutic group being classified as a responder.

### Secondary outcomes

Modified Ashworth Scale (MAS): This scale is designed to measure muscle tone, generally defined as the resistance of muscle as it is being passively lengthened or stretched (Bohannon and Smith, 1987). The muscle groups tested in the upper and lower limbs will be averaged to get a total score. The participant will be placed in the supine position. When testing a muscle that primarily flexes, the joint will be placed in a maximally flexed position and moved to a position of maximal extension over a 1 s count of “one thousand one.” When testing a muscle that primarily extends a joint, the joint will be placed in a maximally extended position and moved to a position of maximal flexion over one second (count “one thousand one”), while grading the ability of the joint to move through the passive range of motion relative to the patient’s muscle tone. The scale is graded as follows:

0: No increase in muscle tone.

1: Slight increase in muscle tone, with a catch and release or minimal resistance at the end of the range of motion when an affected part is flexed or extended.

1+: Slight increase in muscle tone, manifested as a catch, followed by minimal resistance through the remainder (less than half) of the range of motion.

2: A marked increase in muscle tone throughout most of the range of motion, but affected part(s) are still easily moved.

3: Considerable increase in muscle tone, passive movement is difficult.

4: Affected part is rigid in flexion or extension.

### Ancillary outcomes

10 m walk test (10mWT): The 10mWT is used to assess walking speed in meters/s (m/s) over a short distance (Jackson et al., 2008; Bahrami et al., 2017). The start and end point of a clear 10 m walkway will be marked. Additional marks will be added at 2 m and 8 m (identifying the central 6 m to be timed). The total time taken to ambulate 6 m is recorded in m/s. The test will not involve the use of any assisted devices.

Pediatric Evaluation of Disability Inventory Computer Adaptive Test (PEDI-CAT): PEDI-CAT contains item bank of 276 functional activities acquired throughout infancy, childhood and young adulthood (Haley et al., 2011). It will be completed by the parents/caregivers of study participants. The PEDI-CAT measures function in four domains: (1) Daily Activities; (2) Mobility; (3) Social/Cognitive, and (4) Responsibility. The items enable clinicians to construct a description of a child’s current functional status or progress in acquiring routine functional skills.

Gross Motor Function Measure-66 (GMFM66): The GMFM66 consists of a subset of the original 88 items of the GMFM88 divided into five dimensions: Lying and rolling (4 items), sitting (15 items), crawling and kneeling (10 items), standing (13 items), and walking, running, and jumping (24 items) (Beckers and Bastiaenen, 2015). The GMFM66 was developed to improve the interpretability and clinical utility of the GMFM88. A computer program, the Gross Motor Ability Estimator will be used.

Pain Assessment Instrument for CP (PAICP): Nonverbal scales for pain assessment are necessary for CP patients due to their inability to express pain verbally, visual and cognitive impairment. The PAICP instrument consists of 6 drawings of daily situations that are usually not painful and 6 that usually are painful. Patients rate the pain associated with each activity using a Faces Pain Scale (Boldingh et al., 2004).

Vitals including Heart Rate, Blood Pressure and Oxygen Saturation: Vitals will be measured at every visit using Carescape V100 vital signs monitor (GE Healthcare, WI, United States).

### Sample size and power

We assume a true sham responder rate of 0.22 or less, and a treatment responder rate of 0.68 or higher, based upon pilot study observations and assuming 10% attrition of participants during the study. Thus, a sample size of 60 will support 80% power to distinguish between the arms, in a two-sided exact test at the 0.05 significance level. Considering the heterogeneity of prospective participants, proposed sample size of 60 participants (30 per arm) will ensure 80% power for primary endpoint comparison, allowing for precise estimation of responder rates in different demographic subpopulations. Assuming 25% attrition rate of participants in screening based on pilot study, the required number of participants to be screened is 80. The proposed sample size is consistent with other similar studies (NCT05020834, NCT04725019, NCT05154253, NCT05351138).

### Statistical analysis

Randomization will be stratified by age and GMFCS level. Demographics and baseline characteristics will be summarized for all patients using summary statistics for continuous variables (number of patients, mean, standard deviation, median, minimum and maximum) and using group frequencies or percentages for discrete variables. Study arms will be compared with respect to these characteristics using two-sample t-tests, binomial-based tests, chi-squared tests, or nonparametric alternatives. The primary endpoint, the GMFM88 score responder rate (i.e., >5-point increase from baseline), will be estimated and compared between groups using a two-sided chi-squared test at the 0.05 significance level. In the event of small expected cell sizes (e.g., <5), an exact test will be used. If baseline characteristics differ between study arms, logistic regression will be employed, with those characteristics, as well as stratification factors, as covariates. The secondary endpoint, the Modified Ashworth Scale (MAS) responder rate (i.e., > 0.45 change from baseline), will also be estimated and compared between treatment arms using a two-sided chi-squared test. Change-from-baseline to Phase I evaluation in MAS, GMFM88 Dimension A, B, C, D, and E, PEDI-CAT, and 10MWT (GMFCS levels I, II, and III only) will be computed for each participant as the difference between the two measurements; these will be compared between treatment groups using two-sided, two-sample *t*-tests at the 0.05 significance level. Data transformations or nonparametric methods will be substituted if the data are not found to be normally distributed. As an additional ancillary analysis, the Phase I primary, secondary, and ancillary efficacy endpoints will be compared between study arms using logistic regression, analysis of variance, or other statistical modeling appropriate to the endpoint, with stratification factors and other baseline and demographic factors as covariates. Interaction testing and subgroup analyses will be employed to investigate consistency or heterogeneity of treatment effect across subgroups based upon age, gender, race, ethnicity, clinical site, underlying condition, and/or other baseline and demographic factors. The ancillary endpoints will also be estimated in the treatment group as change from baseline to the final evaluation visit (post 8-or 16-week, visit 34), and in the control group as change from the Phase I post 8-week evaluation to the final evaluation visit (post 8 or 16-week, visit 34), with 95% confidence intervals (CI). Similar methods will be employed to characterize change from baseline (as well as change from final evaluation visit) through the follow-up phase (week 24). Safety analyses will establish frequencies of adverse events, in total and by type, along with 90% CIs. Analyses will be conducted under intent-to-treat principles. The extent of missing data will be evaluated and addressed using multiple imputation (subject to evidence supporting an assumption of Missing at Random) together with sensitivity analyses.

## Data management and safety

Parents and/or legal guardians of all eligible participants will be invited to discuss the protocol and its risks and benefits with the site Principal Investigator (PI). Parents and/or legal guardians of potential participants will be given at least 72 h and will be encouraged to read the informed consent, and discuss the study with their child, physician, family, and friends, before signing the IRB-approved informed consent. The informed consent forms will be written in a language understood by an eighth-grade student and will contain information on all outcomes as well as PI’s contact information. IRB-approved age-appropriate assent forms will be provided to minors, based on considerations such as the age, maturity, and degree of literacy. Assent from pre/non-literate children will be obtained in accordance with local IRB requirements.

The original signed informed consent (and assent, if applicable) and two copies will be kept in the PI’s office. No participant will be allowed to enroll without being examined by site PI. The participants will be monitored closely to assess for evidence of complication from experimental protocol. The data monitor, clinical research operations team and medical monitor will closely monitor the participants in regards to the procedures and will be available for consultation.

During training, every participant will be slowly acclimated to the treadmill and other devices. Vitals will be monitored (blood pressure, oxygen saturation, heart rate, pain scores) at each session. Stimulation will be stopped in case of an adverse event during and the participant will receive appropriate standard of care treatment. Before and after every session, a physical therapist will examine the participant’s skin for irritations and abrasions. If skin irritations or abrasions are caused by the recording electrodes or hand placements of trainers, electrode and hand placement will be modified. Furthermore, the therapist will monitor the participant’s skin and muscle for signs of muscle strain, joint sprain, and skin irritation (e.g., temperature and redness). The therapist will assess the appropriate lower extremity parameters and monitor manual assistance by trainers to avoid joint sprain. The physical therapist or trained staff member will stretch the muscles of the participants before and after each training session to prevent injury. PAICP will be completed at every session to monitor for pain signs.

To protect confidentiality, each participant will be assigned a coded identification number for all evaluations and analyses. Data (including video files) will be reported using an electronic data capture system (EDC). Original video files and physical materials will be secured in a locked storage area of the laboratory. Only members of the research team will have access to the data for analyses. SpineX team will meet regularly with the contract research organization.

Adverse events (AE), Adverse Device Effect (ADE) and Device Deficiency (DD) will be reported in a timely manner via electronic CRFs, and will be reported to the Institutional Review Board in accordance with local requirements. Serious Adverse Events (SAE), Series Adverse Device Effect (SADE), and Unanticipated Adverse Device Effects (UADEs) will be reported to IRBs, investigators, and the U.S. FDA in accordance with applicable regulatory requirements. All adverse events will be classified based on their severities as either mild, moderate or severe. Protocol amendments, if applicable, will be approved prior to implementation by overseeing IRBs. An immediate change of protocol will be made in the case of serious, unexpected events. If a change is required, this may be implemented to protect the welfare of the participant prior to obtaining IRB approval. However, every effort will be made to contact the IRB and request approval prior to implementation. Based on our pilot studies (Edgerton et al., 2021; Gad et al., 2021; Hastings et al., 2022), the anticipated adverse events are listed in Table 3.

**Table 3:** Summary of anticipated adverse events.

Adverse event	Likelihood
Skin breakage around the site of stimulation	Rare
Changes in blood pressure	Likely
Skin irritation, discoloration or pain around the site of stimulation	Unlikely
Skin irritation due to placement of trainers’ hand	Likely
Electric shock	Unlikely
Sore muscles	Unlikely
Burns	Unlikely
Tingling of extremities during and after the session	Likely
Increased heart rate	Likely
Light-headedness and dizziness	Rare

## Ethics and dissemination

The trial will be conducted in accordance with the Declaration of Helsinki and the International Conference on Harmonization Good Clinical Practice Guidelines, and applicable regulatory requirements (Goodyear et al., 2007). Central and site level ethical approval will be obtained via the central IRB or local IRBs prior to enrollments. The proposed trial is registered on clinicaltrials.gov (NCT05720208).

Informed consent forms will include language noting that the trial and its results will be shared on ClinicalTrials.gov. Each clinical site will be registered and will provide results to ClinicalTrials.gov in compliance with NIH Policy. The final study report will be submitted to US FDA and all data including study results will be uploaded to the ClinicalTrials.gov database. The results will also be published in relevant scientific journals.

We will undertake initiatives to reach medical professionals external to the trial and introduce them to the application of SCiP™ by virtual and in-person workshops at clinical sites or scientific conferences. Every effort will be made to facilitate implementation and replication of the trial on global scale via our well-established network of collaborations. The long-term goal of this trial is the transition of this novel treatment into clinical practice. The key steps in this process will include: (1) obtaining clinical approval from FDA for SCiP™ as a therapeutic indication to promote motor recovery in children with CP; (2) developing the clinical practice guidelines and reimbursement billing codes for this therapy; and (3) educating medical professionals as well as consumers on the delivery, benefits and any potential side effects of this novel therapy.

## Data availability statement

The original contributions presented in the study are included in the article/Supplementary material, further inquiries can be directed to the corresponding author.

## Author contributions

RS and PG wrote the manuscript. All authors edited the manuscript and provided consent for publication.

## Funding

The authors acknowledge the funding support from BEL13VE in Miracles Jack Jablonski Foundation, Consortium for Technology and Innovation in Pediatrics (CTIP), Brain Recovery Project (BRP), Cerebral Palsy Alliance (Australia) and Cerebral Palsy Alliance Research Foundation (United States).

## Conflict of interest

PG and VE have Shareholder interest in SpineX Inc. VE has shareholder interest in Onward Medical.

The remaining authors declare that the research was conducted in the absence of any commercial or financial relationships that could be construed as a potential conflict of interest.

## Publisher’s note

All claims expressed in this article are solely those of the authors and do not necessarily represent those of their affiliated organizations, or those of the publisher, the editors and the reviewers. Any product that may be evaluated in this article, or claim that may be made by its manufacturer, is not guaranteed or endorsed by the publisher.
