# Supplementary material for: sPinal cOrd neUromodulatioN to treat Cerebral palsy in pEdiatrics: POUNCE Multisite Randomized Clinical Trial
Source: Front Neurosci. 2023 Jul 26;17:1221809. doi: 10.3389/fnins.2023.1221809 (PMC10411340; doi:10.3389/fnins.2023.1221809)
Supplement: Supplementary file 1 [file Table_1.docx]

| **Inclusion Criteria** | All subjects are required to meet the following inclusion criteria in order to be considered eligible for participation in this study:   1. Subject is ≥ 2 and ≤ 13 years old at the time of enrollment/consent. 2. Subject has a diagnosis of cerebral Palsy (CP) classified as Gross Motor Function Classification System (GMFCS) Levels I-V. 3. Minimum score of 12 and Maximum score of 85 on the Gross Motor Function Measure 88 (GMFM-88) total score scale. 4. Subject must have spastic CP hemiplegia or diplegia, quadriplegia. 5. Subject’s medical condition is stable as determined by the investigator. 6. Subject has adequate social support network to be able to participate in training and assessment sessions for the duration of the study, at the discretion of the Investigator. 7. Subject is capable of performing simple cued motor tasks and can follow 2-3 step commands. 8. Subject can communicate an accurate yes or no answer to questions according to parents. |
| --- | --- |
| **Exclusion Criteria** | Subjects will be excluded from participating in this study if they meet any of the following exclusion criteria:   1. Subject has a concurrent neurological disease affecting the central nervous system. 2. Subject has implanted stimulator (e.g., epidural stimulator, vagus nerve stimulator, pacemaker, cochlear implant, etc.) or drug delivery device (e.g., baclofen pump) 3. Subject is dependent on an electro-magnetic medical implant (e.g., cardiac pacemaker or implanted drug pump), ventilation support, or another external device. 4. Subject has received botulinum toxin injection within 6 months preceding enrollment. 5. Subject is unable to participate in activity based neurorehabilitation therapy (ABNT) without orthosis. 6. Subject has limited life expectancy or co-morbid conditions, social/psychological problems, or cognitive impairments that, in the opinion of the investigator, will preclude them from participation and completion of study procedures or requirements. 7. Subject has a medical condition or complications related to the use of certain medications that may affect validity of the study as determined by the investigator. 8. Subject has a medical condition not listed above that may put the subject at risk as determined by the investigator. 9. Subject is participating in or plans to participate in another research study that may interfere with study endpoints. 10. Subject is known or suspected to be non-compliant; and/or subject is unable or unwilling to comply with study requirements. 11. Subject has cardiovascular or musculoskeletal disease or injury that would prevent full participation in physical therapy intervention 12. Subject has a history of uncontrolled seizures. 13. Subject has unhealed fracture or other musculoskeletal impairment that might interfere with upper or lower extremity rehabilitation or testing activities. 14. Subject has a history of orthopedic surgery in upper or lower extremities or neurosurgery that may be a confounding factor for interpretation of the results (such as tendon transfer, tendon or muscle lengthening for spasticity management, injection therapies to lower extremity muscles, percutaneous lengthening, spinal fusion etc.) in last 12 months. 15. Subject has a true leg length discrepancy greater than 2 cm. 16. Subject has established osteoporosis and taking medication for osteoporosis treatment. 17. Subject has undergone selective dorsal rhizotomy surgery. 18. Subject has hip or shoulder migration percentage > 30% on anteroposterior radiographic imaging. 19. Subject has less than 20 degrees in hip Range of Motion (ROM) in hip abduction. 20. Subject has unexplained presence of persistent complaints of pain of any kind. 21. Subject has fixed upper or lower extremity contractures of 20 degrees or more. 22. Subject has a fixed spinal scoliosis greater than 20 degrees. 23. Subject has severe cortico-visual impairment. 24. Subject has other ongoing, or who has discontinued less than 14 days prior to consent, physical and occupational therapies including but not limited to robotic therapy, gait training, aqua therapy, hippo therapy, intensive therapies, whole body vibration (WBV), stimulation therapies (e.g., E-Stimulation, Functional Electrical Stimulation (FES), Neuromuscular Electrical Stimulation (NMES)). |

Supplemental table 1: Participant inclusion and exclusion criteria
